# Sodium tanshinone IIA sulfate adjunct therapy reduces high-sensitivity C-reactive protein level in coronary artery disease patients: a randomized controlled trial

**DOI:** 10.1038/s41598-017-16980-4

**Published:** 2017-12-12

**Authors:** Siming Li, Yang Jiao, Hanjay Wang, Qinghua Shang, Fang Lu, Li Huang, Jiangang Liu, Hao Xu, Keji Chen

**Affiliations:** 1 Cardiovascular Diseases Center, Xiyuan Hospital, China Academy of Chinese Medical Sciences, Beijing, 100091 China; 2 Graduate School, Beijing University of Chinese Medicine, Beijing, 100029 China; 3 Department of Cardiothoracic Surgery, Stanford University, Palo Alto, CA 94304 USA; 4 Institute of Clinical Pharmacology, Xiyuan Hospital, China Academy of Chinese Medical Sciences, Beijing, 100091 China; 5 Integrative Cardiology Department, China-Japan Friendship Hospital, Beijing, 100029 China

## Abstract

High-sensitivity C-reactive protein (hs-CRP) is independently associated with cardiovascular events in coronary artery disease (CAD) patients and reducing the hs-CRP level may further benefit this population. We conduct this parallel design, randomized-controlled trial to assess the effectiveness of adjunct sodium tanshinone IIA sulfate (STS) therapy on circulating inflammation markers in CAD patients. Unstable angina or non-ST-elevation myocardial infarction patients with increased hs-CRP level were randomly assigned to atorvastatin-based standard medical therapy or standard therapy plus STS injection (80 mg, once daily for 14 consecutive days). The primary outcome was hs-CRP level. After the 14-day treatment, the experimental group (n = 35) exhibited significantly lower levels of hs-CRP than the control group (n = 35) (1.72 vs 3.20 mg/L, p = 0.0191). Lower levels of interleukin-6, monocyte chemotactic protein-1 (MCP-1), and soluble CD40 ligand were also observed in the experimental group. Angina symptoms were also better controlled in the experimental group. At 30 days after treatment completion, MCP-1 levels remained lower in the experimental group than in the control group (313.88 vs 337.91 pg/mL, p = 0.0078). No serious adverse events occurred. Our study demonstrates that on the basis of standard medical therapy, STS further reduce elevated hs-CRP and other circulating inflammation markers in CAD patients. (Chictr.org number: ChiCTR-TRC-12002361).

## Introduction

Cardiovascular disease is the worldwide leading cause of death, and coronary artery disease (CAD) is responsible for the greatest mortality. Inflammation plays an important role both in the development of atherosclerosis and in triggering cardiovascular events^1^. As a robust marker of systematic inflammation, high-sensitivity C-reactive protein (hs-CRP) has been closely studied^2–5^, and a meta-analysis showed that hs-CRP >3 mg/L is an independent risk factor for cardiovascular events^6^. Therefore, reducing the concentration of hs-CRP may further benefits patients with CAD. In fact, the mortality reduction and cardiovascular benefits of statins, a cornerstone of evidence-based standard medical therapy for CAD, may partly attribute to its anti-inflammation effect.

Indeed, the JUPITER trial found that, among non-hyperlipidemic patients with elevated hs-CRP, statin therapy reduced hs-CRP levels by 37%, and led to a significantly lower rate of major adverse cardiovascular events after 1.9 years compared to placebo^7^. Nevertheless, despite intensive statin therapy, 22.4% of patients with acute coronary syndrome suffer a serious cardiovascular or cerebrovascular event within two years of initiating treatment^8^, thus revealing the extent of residual cardiovascular risk, as well as the potential benefit of further dampening the inflammatory reaction of atherosclerosis.

**Figure 1:**
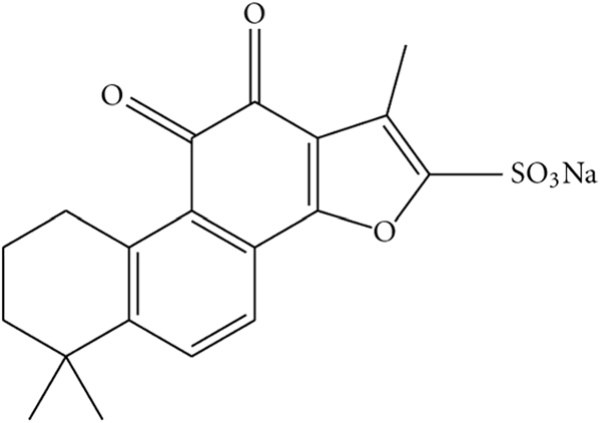
The chemical structure of sodium tanshinone IIA sulfate.

Tanshinone IIA, one of the most pharmacologically active components extracted from *Radix Salviae miltiorrhizae*, has been identified as a promising natural cardioprotective agent^9^. Most noteworthy is the ability of tanshinone IIA to decrease the levels of multiple inflammatory factors associated with the progression of atherosclerosis, such as CRP, interleukin-6 (IL-6), tumor necrosis factor alpha (TNF-𝛼), vascular cell adhesion molecule-1 (VCAM-1), CD40, monocyte chemotactic protein-1 (MCP-1), and matrix metalloproteinase-9 (MMP-9)^10,11^. Because of the low oral bioavailability of tanshinone IIA^12^, intravenous sodium tanshinone IIA sulfate (STS) has been developed (Fig. 1), and is the most widely used clinical formulation of tanshinone IIA in China for patients with CAD.

In this trial, we hypothesized that the addition of STS to standard statin-containing medical therapy for patients with CAD will: 1) further reduce the levels of serum hs-CRP and other circulating inflammatory markers after 14 days of treatment; 2) show a sustained effect on inflammatory marker levels at 30 days after treatment completion; 3) improve angina symptoms; and 4) be a safe treatment, compared to standard medical therapy alone.

## Results

### Participant characteristics

**Figure 2:**
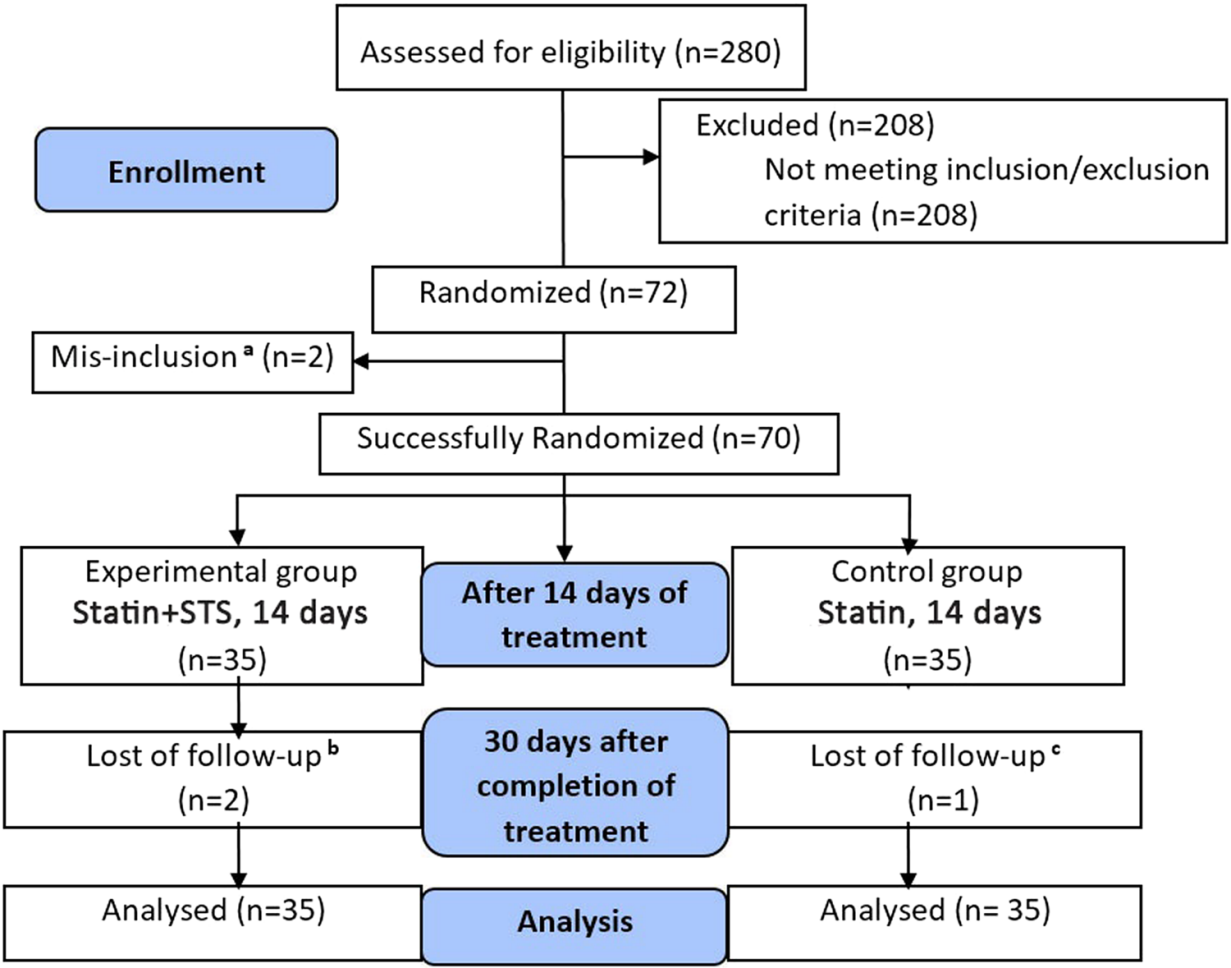
Flow Diagram of Study Design. ^a^Two patients were excluded because of mis-inclusion: 1 patient in the control statin group elected to undergo percutaneous coronary intervention during hospitalization, and 1 patient in the experimental statin +STS group developed pneumonia. ^b^Two patients were lost to follow up in the experimental statin +STS group, due to inability to contact the patients for data collection. ^c^One patient was lost to follow up in the control statin group after the patient elected to withdraw from the study. STS, sodium tanshinone IIA sulfate.

Study participants were recruited from August 2012 to January 2014, and final follow-up was completed by February 2014. A flow diagram illustrating the study design is shown in Fig. 2. A total of 280 inpatients were assessed for eligibility, and finally, 72 eligible participants were enrolled. Among which, 36 participants were randomly assigned to receive standard statin-containing medical therapy with uniformly-dosed atorvastatin 20 mg daily (control statin group) and 36 to standard medical therapy plus 80 mg intravenous STS daily (experimental statin+STS group). Two patients were subsequently excluded because of mis-inclusion: 1 patient in the statin group withdrew to receive percutaneous coronary intervention (PCI) during hospitalization, and 1 patient in the statin+STS group developed pneumonia. Ultimately, 70 participants (35 in each group) completed the 14-day treatment. During the 30-day follow-up period, 3 participants, 2 in the statin+STS group and 1 in the statin group, were lost to follow up. Among them, one declined to participate in the follow-up since she has left Beijing, two were unreachable for data collection. Intention-to-treat analysis was performed, involving all 70 patients who were successfully randomized, and the last-observation-carried-forward (LOCF) imputation method was used to account for missing data.

Of our 70 enrolled patients, 63 (90%) presented with unstable angina, and 7 (10%) presented with non-ST-elevation myocardial infarction (NSTEMI). CAD diagnosis was documented by previous coronary angiography performed any time prior to enrollment in 57 patients (81.4%), and via prior or present history of myocardial infarction in 13 patients (18.6%). The baseline characteristics of the participants are shown in Table 1, and the two groups were comparable in demographics, presenting diagnosis, cardiovascular risk factors, number of diseased vessels, thrombolysis in myocardial infarction (TIMI) risk score, and blood lipid concentrations. The use of other cardiovascular medications comprising standard medical therapy for CAD, including aspirin, clopidogrel, beta-blockers, angiotensin-converting enzyme inhibitors (ACEI), angiotensin receptor blockers (ARB), calcium channel blockers (CCBs), and agents for diabetes mellitus, was also similar between the groups.

**Table 1:** Baseline Characteristics.

Baseline Characteristics	Experimental group: Statin + STS (n = 35)	Control group: Statin (n = 35)
Demographics		
Male, N(%)	18 (51.4)	20 (57.1)
Age, mean ± SD	66 ± 7.28	67.47 ± 6.52
Diagnosis		
NSTEMI, N(%)	3 (8.6)	4 (11.4)
UA, N(%)	32 (91.4)	31 (88.6)
Number of diseased vessels, N(%)		
One	11 (31.4)	8 (22.9)
Two	6 (17.1)	6 (17.1)
three	4 (11.4)	8 (22.9)
Cardiovascular risk factors, N(%)		
Hypertension	27 (77.1)	33 (94.3)
Hyperlipidemia	26 (74.3)	30 (85.7)
Diabetes mellitus	17 (48.6)	13 (37.1)
Smoking history	9 (25.7)	8 (22.9)
BMI (mean ± SD, kg/m^2^)	24.47 ± 2.89	24.86 ± 3.72
TIMI risk score, N(%)		
High risk (score 5–7)	2 (2.9)	3 (4.3)
Middle risk (score 3–4)	21 (30.0)	20 (28.6)
Low risk (score 0–2)	12 (17.1)	12 (17.1)
Medication, N(%)		
Beta-blocker	21 (60.0)	24 (68.6)
ACEI	9 (25.7)	12 (34.3)
ARB	10 (28.6)	10 (28.6)
CCB	17 (48.6)	19 (54.3)
Aspirin	34 (97.1)	29 (82.9)
Clopidogrel	17 (48.6)	24 (68.6)
Blood lipid (mean ± SD, mmol/L)		
TC	4.39 ± 1.28	4.22 ± 1.29
TG	1.71 ± 0.80	1.95 ± 0.92
LDL	2.73 ± 0.99	2.66 ± 0.85

All comparisons between the groups were not significantly different (p > 0.05 for all). ACEI, angiotensin-converting enzyme inhibitor; ARB, angiotensin receptor blocker; BMI, body mass index; CCB, calcium channel blocker; IQR, interquartile range; LDL, low density lipoprotein; NSTEMI, non-ST segment elevation myocardial infarction; SD, standard deviation; TC, total cholesterol; TG, triglyceride; TIMI, Thrombolysis in Myocardial Infarction; UA, unstable angina.

### Primary endpoint

**Figure 3:**
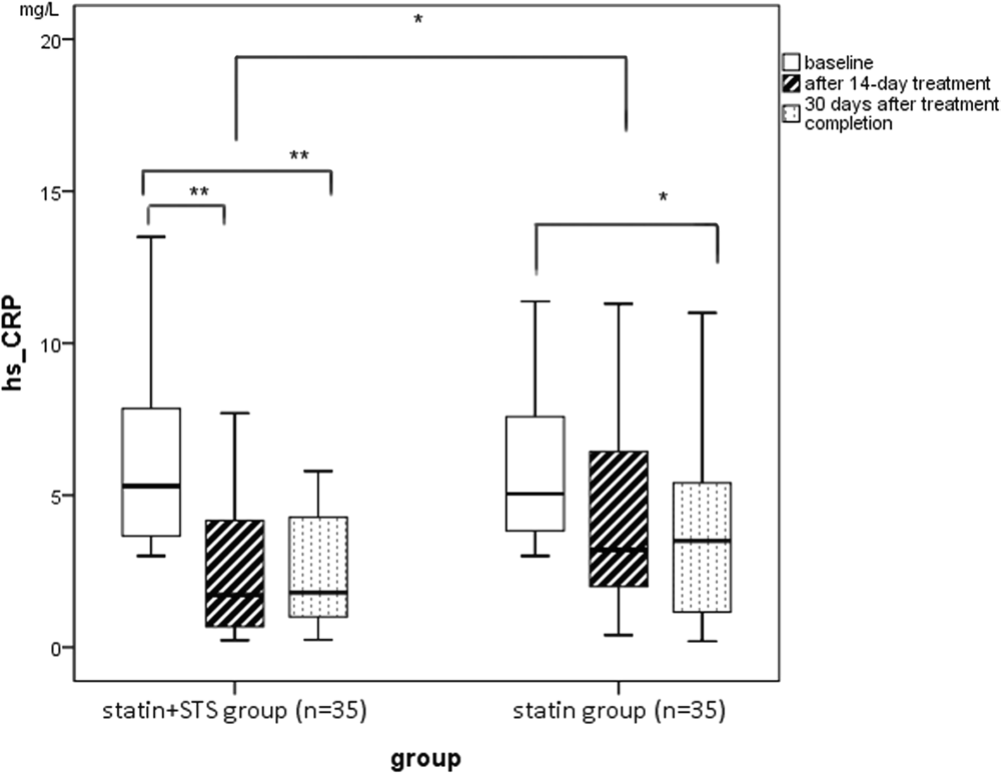
Comparison of hs-CRP level between groups at baseline, after 14-day treatment, and at 30 days after completion of treatment. *Statistically significant, p < 0.05. **Statistically significant, p < 0.01. hs-CRP, high-sensitivity C-reactive protein; STS, sodium tanshinone IIA sulfate.

The levels of hs-CRP in the experimental statin + STS group and control statin group at baseline, immediately after 14 days of treatment, and at 30 days after completion of treatment are illustrated in Fig. 3. The data for comparisons in hs-CRP level between the statin + STS and statin groups are delineated in Table 2, while the data for comparisons within each group are presented in Table 3.

**Table 2:** Comparison of hs-CRP between groups at baseline and after treatment.

Primary outcome: hs-CRP, median (IQR)	Experimental group: Statin + STS (n = 35)	Control group: Statin (n = 35)	P value
Baseline:			
hs-CRP, mg/L	5.35 (3.58, 8.00)	5.04 (3.81, 7.60)	0.3894
After 14-day treatment:			
hs-CRP, mg/L	1.72 (0.64, 4.24)	3.20 (1.99, 6.83)	0.0191
Absolute hs-CRP reduction, mg/L	2.90 (1.30, 6.05)	1.54 (−1.45, 3.51)	0.0485
Percent hs-CRP reduction, %	71.75 (24.07, 87.70)	30.00 (−22.14, 63.58)	0.0114
30 days after treatment completion:			
hs-CRP, mg/L	1.80 (0.90, 4.30)	3.50 (1.11, 5.60)	0.2408
Absolute hs-CRP reduction, mg/L	2.74 (0.86, 5.16)	1.38 (−0.25, 3.93)	0.1963
Percent hs-CRP reduction, %	69.14 (18.18, 84.44)	30.00 (−8.19, 77.98)	0.2309

hs-CRP, high-sensitivity C-reactive protein; IQR, interquartile range; STS, sodium tanshinone IIA sulfate. Inter-group comparison was analyzed using the Wilcoxon rank-sum test, due to non-normal distribution of the data.

**Table 3:** Comparison of hs-CRP, IL-6, MCP-1, sCD40L, MMP-9, sVCAM-1, TNF-α levels within each group at baseline and after treatment.

Inflammatory Markers, Median (IQR) or Mean ± SD	Baseline	After 14-day treatment	30 days after treatment completion	△1	P value^1^	△2	P value^2^
hs-CRP, mg/L^b^							
Statin + STS group	5.35 (3.58, 8.00)	1.72 (0.64, 4.24)	1.80 (0.90, 4.30)	−2.83	0.00	−2.81	0.00
Statin group	5.04 (3.81, 7.60)	3.20 (1.99, 6.83)	3.50 (1.11, 5.60)	−1.01	0.26	−1.95	0.03
IL-6, pg/mL^b^							
Statin + STS group	14.58 (13.39, 18.45)	7.76 (5.98, 10.74)	9.30 (5.66, 13.11)	−6.30	0.00	−6.01	0.00
Statin group	15.18 (12.50, 18.15)	9.55 (8.35, 11.92)	8.81 (7.17, 11.33)	−3.67	0.00	−5.58	0.00
MCP-1, pg/mL^b^							
Statin + STS group	369.79 ± 36.24	301.65 ± 33.75	313.88 ± 37.91	−66.87	0.00	−53.06	0.00
Statin group	368.52 ± 37.18	327.24 ± 30.65	337.91 ± 27.62	−41.39	0.00	−30.71	0.00
sCD40L, pg/mL^a^							
Statin + STS group	934.48 ± 313.16	728.80 ± 196.68	853.19 ± 246.98	−215.71	0.00	−88.01	0.03
Statin group	941.03 ± 260.95	848.90 ± 267.89	878.73 ± 245.20	−93.88	0.02	−59.63	0.13
MMP-9, ng/mL^a^							
Statin + STS group	38.88 (23.73, 48.01)	17.61 (15.82, 20.11)	18.5 (14.17, 24.33)	−19.99	0.00	−19.43	0.00
Statin group	35.07 (23.73, 42.73)	19.22 (16.36, 24.92)	20.65 (16.25, 28.22)	−12.92	0.00	−13.46	0.00
sVCAM-1, ng/mL^a^							
Statin + STS group	807.25 ± 208.21	797.24 ± 242.28	753.27 ± 177.80	−74.39	0.01	−56.16	0.06
Statin group	731.76 ± 167.57	723.42 ± 216.73	745.08 ± 195.32	−68.24	0.02	−43.71	0.14
TNF-α, pg/mL^b^							
Statin + STS group	20.25 ± 7.99	14.26 (10.69, 19.05)	14.72 (10.24, 19.94)	−5.29	0.00	−4.20	0.00
Statin group	20.90 ± 10.03	13.99 (9.88, 30.15)	15.56 (10.97, 21.61)	−3.41	0.00	−4.43	0.00

hs-CRP, high-sensitivity C-reactive protein; IL-6, interleukin-6; IQR, interquartile range; MCP-1, monocyte chemotactic protein-1; MMP-9, matrix metalloproteinase-9; sCD40L, soluble CD40 ligand; sVCAM-1, soluble vascular cell adhesion molecule-1; SD, standard deviation; STS, sodium tanshinone IIA sulfate; TNF-𝛼, tumor necrosis factor alpha.

△1 indicates the intra-group difference between baseline and immediately after 14-day treatment.

△2 indicates the intra-group difference between baseline and at 30 days after the completion of treatment.

^1^p-value for intra-group comparisons between baseline and after 14-day treatment.

^2^p-value for intra-group comparisons between baseline and at 30 days after the completion of treatment.

^a^Intra-group comparison was analyzed using student’s t-test, due to normal distribution of the data.

^b^Intra-group comparison was analyzed using the Wilcoxon rank-sum test, due to non-normal distribution of the data.

Baseline hs-CRP level was similar in the statin+STS and statin groups (5.35 vs 5.04 mg/L, respectively, p = 0.3894). After 14 days of treatment, however, hs-CRP level was significantly lower in the statin+STS group than in the statin group (1.72 vs 3.20 mg/L, p = 0.0191). Indeed, the magnitude of the absolute and relative reductions in hs-CRP level after 14 days of treatment were greater for the statin+STS group than for the statin group. Whereas a significant reduction in hs-CRP level was observed within the statin+STS group after 14 days of treatment compared to baseline (1.72 vs 5.35 mg/L, p < 0.01), the change in hs-CRP level within the statin group was not significant (3.20 mg/L after 14 days of treatment vs 5.04 mg/L at baseline, p = 0.26).

At 30 days after the completion of treatment, hs-CRP levels in the statin+STS and statin groups were not significantly different (1.80 vs 3.50 mg/L, respectively, p = 0.2408). The level of hs-CRP within the statin+STS group, however, remained significantly lower at 30 days after treatment completion than at baseline (1.80 vs 5.35 mg/L, p < 0.01). The level of hs-CRP within the statin group, too, was significantly lower at 30 days after treatment completion than at baseline (3.50 vs 5.04 mg/L, p = 0.03).

In addition, the reduction of the experimental group was more significant than that of the control group (2.99 vs 1.54 mg/L, P = 0.0485) after 14-day treatment, so as the percent change (71.75% vs 30%, P = 0.0114) (Table 2).

### Secondary endpoints

#### Other circulating inflammatory marker levels

**Figure 4:**
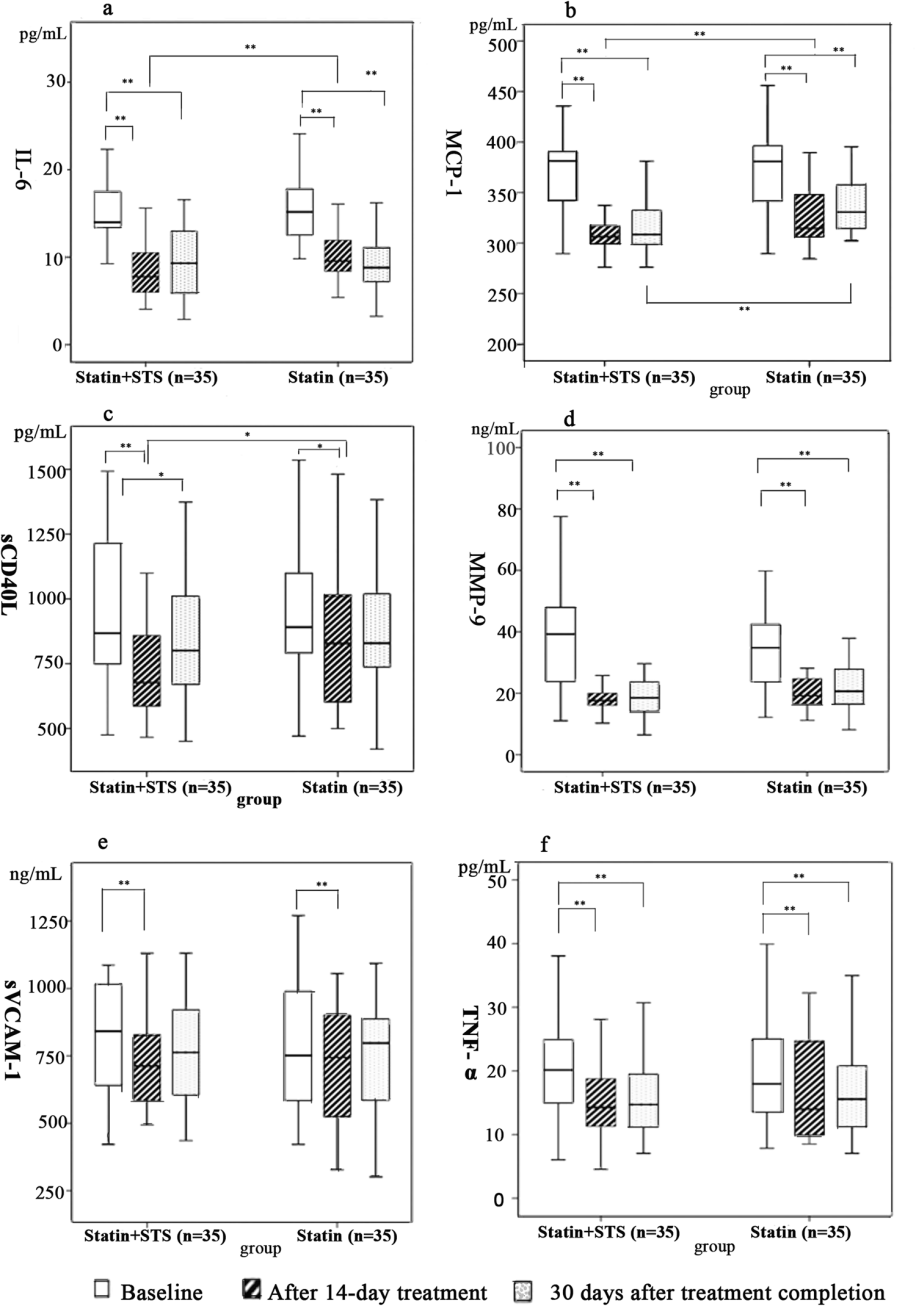
Comparison of IL-6, MCP-1, sCD40L, MMP-9, sVCAM-1, and TNF-𝛼 levels between groups at baseline, after 14-day treatment, and at 30 days after completion of treatment. *Statistically significant, p < 0.05. **Statistically significant, p < 0.01. IL-6, interleukin-6; MCP-1, monocyte chemotactic protein-1; MMP-9, matrix metalloproteinase-9; sCD40L, soluble CD40 ligand; sVCAM-1, soluble vascular cell adhesion molecule-1; STS, sodium tanshinone IIA sulfate; TNF-𝛼, tumor necrosis factor alpha.

The levels of IL-6, MCP-1, soluble CD40 ligand (sCD40L), MMP-9, soluble VCAM-1 (sVCAM-1), and TNF-α in the experimental statin + STS group and control statin group at baseline, immediately after 14 days of treatment, and at 30 days after completion of treatment are illustrated in Fig. 4. The data for comparisons within each group are presented in Table 3.

For all the inflammatory markers above, baseline levels were similar between the 2 groups. After 14 days of treatment, the level of IL-6 in the statin + STS group was significantly less than that in the statin group (7.76 vs 9.55 pg/mL, p = 0.0096), and the same was true of MCP-1 (301.65 vs 327.24 pg/mL, p = 0.0014) and sCD40L (728.80 vs 848.90 pg/mL, p = 0.0361). In addition, within both groups, all of the inflammatory markers were significantly decreased after 14 days of treatment compared to baseline.

At 30 days after the completion of treatment, the level of MCP-1 in the statin + STS group remained significantly less than that in the statin group (313.88 vs 337.91 pg/mL, respectively, p = 0.0078). Whereas levels of all the other inflammatory markers were similar between the 2 groups. Within the statin + STS group, the levels of all the inflammatory markers except sVCAM-1 remained significantly lower at 30 days after treatment completion than at baseline, and within the statin group, the same was true for all the inflammatory markers except sCD40L and sVCAM-1.

No significant differences in the levels of MMP-9, sVCAM-1, and TNF-α were observed between the 2 groups after 14 days of treatment, or at 30 days after the completion of treatment.

#### Angina

Total angina scores for the experimental statin + STS group and control statin group at each time point are presented in Table 4. The median total angina score (no angina = 0 points, maximum = 24 points) was similar at baseline between the statin+STS and statin groups (10 vs 12 points, respectively, p = 0.105). Median total angina score for the statin+STS group was significantly lower compared to that of the statin group both immediately after 14 days of treatment (0 vs 6 points, respectively, p < 0.01) and at 30 days after the completion of treatment (2 vs 8 points, p < 0.01).

**Table 4:** Comparison of angina score between groups at baseline and after treatment.

Total Angina Score, median (IQR)	Experimental group: Statin + STS (n = 35)	Control group: Statin (n = 35)	P value
Baseline	10 (6, 12)	12 (8, 14)	0.105
After 14-day treatment	0 (0, 6)	6 (0, 10)	0.00
30 days after treatment completion	2 (0, 8)	8 (6, 10)	0.00

IQR, interquartile range; STS, sodium tanshinone IIA sulfate. Inter-group comparison was analyzed using the Wilcoxon rank-sum test, due to non-normal distribution of the data.

#### Safety

No significant adverse side effects (e.g. bleeding events, abnormal liver or kidney function tests) or major adverse cardiovascular events (e.g. myocardial infarction, stroke, urgent coronary revascularization, death) were observed during the whole study period in all 72 participants. Two participants in the statin + STS group complained of mild dizziness (n = 1) or headache (n = 1) with initiation of treatment, which all fully disappeared the next day without special intervention. At 30 days after the completion of treatment, the survival in both groups was 100%.

## Discussion

Recent research has established a fundamental role for inflammation in atherosclerosis and CAD. Among the various systemic inflammatory markers, hs-CRP has the greatest predictive value for atherosclerotic plaque stability and acute cardiovascular events^5^. In the JUPITER trial, *de novo* initiation of rosuvastatin 20 mg daily reduced the incidence of major adverse cardiovascular events in apparently healthy persons without hyperlipidemia but with elevated hs-CRP level^7^, thus suggesting the potential of hs-CRP to be a therapeutic target. A meta-analysis including 22 trials indicated that hs-CRP >3 mg/L was independently associated with risk of incident CAD events^6^, while hs-CRP >15 mg/L may be more suggestive of infection. Therefore, hs-CRP was adopted as the primary outcome measure in this trial, but only patients with confirmed CAD and with elevated hs-CRP level between 3 mg/L and 15 mg/L were enrolled. Our results showed that 14 days of adjunctive STS therapy was more effective in reducing elevated hs-CRP level than standard medical therapy alone.

In addition to affecting hs-CRP, adjunctive STS therapy also reduced the levels of IL-6, MCP-1, and sCD40L further than did standard medical therapy alone. IL-6 is a potent pro-inflammatory cytokine that coordinates the release of CRP during the acute-phase response, and may have autocrine, paracrine, and endocrine mechanisms that all contribute to CAD pathogenesis^13^. Furthermore, MCP-1 plays an important role in atherosclerosis as a key chemokine regulating the migration and infiltration of mononuclear cells and macrophages^14^, and CD40-mediated signaling has been implicated in the process of plaque destabilization^15^. The upregulation of IL-6, MCP-1, and CD40L in the setting of CAD has each been associated with worse clinical outcomes^16–19^, and as such, these important inflammatory factors represent additional targets for future anti-inflammatory CAD therapy. However, our study did not show a significant effect of adjunctive STS on levels of TNF-α, MMP-9, and sVCAM-1, which differs from the results of previous experimental studies^10^. Nevertheless, STS represents a promising adjunctive therapy for mitigating residual cardiovascular risk secondary to inflammation in CAD patients. Indeed, Robertson *et al*. recently conducted a large-scale screening of anti-inflammatory compounds using zebrafish as an animal model, and identified tanshinone IIA as one of the most potent anti-inflammatory agents among thousands of compounds screened^20^.

Since STS is an intravenous preparation not suitable for long-term application, we investigated whether adjunctive STS therapy might yield sustained anti-inflammatory effects after discontinuation of treatment. Our results showed that the significant reduction in MCP-1 level following administration of integrative statin-STS therapy compared to standard medical therapy alone persisted to 30 days after the treatment was completed. Although there was no statistically significant difference in hs-CRP reduction between the groups at 30 days after the completion of treatment, we nevertheless observed a clear trend toward lower hs-CRP levels in the statin-STS group as compared to the control group. This negative result might be due to the small sample size of this preliminary trial. Larger clinical trials with longer duration of follow-up are necessary to clarify whether there is a sustained, long-term effect of adjunctive STS on hs-CRP, and whether this promising anti-inflammatory effect might be translatable into clinical benefits.

In addition to anti-inflammatory effects, STS also possesses both anti-platelet and anticoagulant properties^21,22^. Considering the potential for drug interactions between STS and the various anti-thrombotic agents commonly used in conventional CAD treatment, increased hemorrhagic events associated with adjunctive STS therapy may be an issue of concern in clinical practice. In this short-term, small-sized clinical trial, we did not observe any bleeding events or any other serious adverse events associated with adjunctive STS therapy. Our results suggest that the addition of STS to standard medical therapy for CAD might not increase the risk of bleeding events or hepatorenal side effects, although the full safety profile of this integrative therapy should be further assessed in future larger scale clinical trials.

Some limitations of this trial should be noted. First, the sample size was small, and thus the study may not have sufficient power to detect statistical differences between the 2 groups for each serum inflammatory marker. Second, the open-label design of our study introduces the potential for patients, physicians, or researchers to be influenced by their knowledge of a patient’s treatment allocation. Nevertheless, blinded end-point assessment by outcome adjudicators, laboratory blood technicians, and data analysts minimized the potential biases. Third, the short 30-day follow-up period after the completion of treatment limits our ability to resolve the long-term effects of STS on serum inflammatory markers, and also hinders our ability to determine the potential benefit of STS in preventing recurrent cardiovascular events.

In conclusion, we demonstrate in this trial that an 80 mg intravenous dose of STS administered daily for 14 days, as an adjunct to standard medical therapy with uniformly-dosed atorvastatin, further reduced the levels of circulating inflammatory markers including hs-CRP, IL-6, MCP-1, and sCD40L in CAD patients compared to standard medical therapy alone. The apparent additive effect of STS in reducing the level of MCP-1 even persisted to 30 days after the completion of treatment. Our study also demonstrated a considerable improvement in angina symptoms, including the frequency and severity of angina pectoris, as compared with standard medical therapy alone. Given that no previous high-quality study has examined the effect of STS on hs-CRP and other inflammatory markers in CAD patients, our findings shed light on the benefit and safety of an integrative statin + STS regimen for CAD patients with enhanced inflammatory reaction, thereby offering potential implications for clinical practice. Whether the additional reduction of inflammatory factors in CAD patients will ultimately reduce future cardiovascular events and yield long-term prognostic benefit will be the focus of future clinical trials.

## Methods

### Ethics

The study protocol (2012XL022-2) was approved by the ethics review board of Xiyuan Hospital, China Academy of Chinese Medical Sciences (CACMS), and is available^23^. The study is registered at http://www.iecrf.org (Chictr.org number: ChiCTR-TRC-12002361, registered on 07/22/2012). All aspects of our study were conducted with adherence to the current version of the Declaration of Helsinki, the guidelines established by the International Conference on Harmonization of Good Clinical Practice, and the laws of China. All participants signed informed consent forms before enrollment.

### Trial design and settings

This trial was a parallel-group, prospective randomized open-label blinded-endpoint (PROBE) study conducted in China. The eligible participants were randomized 1:1 into either the control group (standard medical therapy for CAD with uniformly-dosed atorvastatin) or the experimental group (control regimen plus 80 mg intravenous dose of STS daily). Because atorvastatin reduces hs-CRP to a greater extent than other statins^24^, and is more popular in clinical practice, we treated participants with uniformly-dosed atorvastatin instead of simvastatin, as originally proposed in our published study protocol.

### Participants

Hospitalized patients with unstable angina or non-ST-elevation myocardial infarction between 35 and 75 years of age were eligible if they were on statin therapies for at least 1 month, had increased hs-CRP level (between 3 mg/L and 15 mg/L) at enrollment, and with documented CAD (with at least 1 coronary artery stenosis ≥50% confirmed by previous coronary angiography, or with a documented history of myocardial infarction [coronary angiography not required]). The diagnosis of CAD was based on the standardized criteria established in “Nomenclature and criteria for diagnosis of ischemic heart disease,” a joint report published by the International Society and Federation of Cardiology and the World Health Organization^25^, and the ESC Guidelines for the management of acute coronary syndromes in patients presenting without persistent ST-segment elevation^26^.

Exclusion criteria included severe heart failure (ejection fraction <35%), reduced platelet count or other bleeding diatheses, cancer, sexually transmitted diseases, tuberculosis, rheumatoid arthritis or other autoimmune diseases, infection, fever, trauma, burn injury, surgery within one month prior to recruitment, or any history of serious pulmonary, hepatic, renal, neurological, psychiatric, or hematological diseases. Additional exclusion criteria included active use of any antibiotics or any traditional Chinese medicine with the function of clearing heat and removing toxins (e.g. Honeysuckle, Forsythiae Fructus, Taraxacum, Rheum officinale, Polygonum cuspidatum). Patients participating in other clinical trials were also excluded. Finally, patients must not have previously (within one month) undergone or currently be planning to undergo PCI or coronary artery bypass grafting, considering their effect on inflammatory factors.

Recruitment, intervention, and data collection took place at the inpatient department of Xiyuan Hospital, CACMS, and China-Japan Friendship Hospital in Beijing, China. Subjects were recruited from August 2012 to January 2014, and follow-up was completed by February 2014.

### Interventions

Each patient in the control group received 20 mg atorvastatin orally once per evening, in addition to any other oral or intravenous medications (e.g. beta-blockers, ACEI/ARB, CCB, aspirin, clopidogrel, nitrates) deemed appropriate for a standard treatment of the patient’s individual condition. Doses of ACEI/ARB or beta-blockers were titrated gradually to target levels whenever possible. These standard medications were prescribed by physicians who were not associated with the trial. Additionally, patients in the experiment group received intravenous STS (80 mg, once daily for 14 consecutive days, 10 mg per ampoule, Jiangsu Carefree Pharmaceutical Co., Ltd., national drug approval number: H31022558), diluted with 250 mL 0.9% sodium chloride solution.

All study participants received treatment as indicated above for 14 consecutive days. After 14 days, patients in both groups continued to receive guideline-based standard medical therapy for CAD.

### Outcome measures

Serum hs-CRP level was the primary outcome. Secondary outcomes included 1) the levels of other inflammatory mediators, including IL-6, TNF-𝛼, VCAM-1, sCD40L, MCP-1, and MMP-9, as measured by enzyme-linked immunosorbent assay; 2) the extent of improvement in angina symptoms; and 3) treatment safety.

The extent of improvement in angina symptoms was evaluated by a scoring system (Supplementary Table S1) based on the frequency, duration, and intensity of angina episodes, as well as the dose of nitroglycerin utilized, according to “Cardiovascular Drug Clinical Research Guiding Principles” by the Ministry of Health, People’s Republic of China, 1998. The total angina score ranges from a minimum 0 points (no angina) to a maximum 24 points, and higher score indicates more severe angina symptoms.

To assess treatment safety, patients were asked to report any side effects or changes in feelings that they had noticed. In addition, the results of routine blood, urine, and stool tests, liver and kidney function tests, coagulation tests, and electrocardiogram tests were also considered in the evaluation of safety. Finally, adverse events including death, adverse cardiovascular events (myocardial infarction, stroke, etc.) were closely monitored and recorded by the clinical researchers during the whole study period.

All outcomes were measured at baseline, immediately after completion of the 14-day treatment, and at 30 days after completion of treatment. No changes to the designated trial outcomes were made after the study commenced.

### Sample size estimation

Because the data needed to perform an *a priori* sample size calculation for this pilot study was not available, we adopted the sample size of a comparable trial which involved 60 subjects randomized into two groups of 30 each^27^. For our study, planned to recruit 72 patients, assuming conservatively that 20% of the participants would be lost to follow-up. No interim analyses were undertaken.

### Randomization and blinding

A member of the Institute of Clinical Pharmacology (ICP) of Xiyuan Hospital who was independent of the study used SAS 9.3 software (SAS Institute, Cary, NC, USA) to perform a permuted-block randomization, generating a sequence of 72 random numbers. To maintain concealment, the group assignments were relayed by another member of the ICP using a central randomization strategy. Once a patient met all the criteria for enrollment in the trial, the group assignment was delivered by telephone to the clinical researchers. Given that the color of the STS solution is unique and challenging to emulate, unblinding was permitted at the physician and patient levels only after baseline data was collected. In order to minimize biases as much as possible, all other potential sources of information that may reveal treatment allocation to the patients were judiciously guarded. Additionally, the details of treatment allocation were not disclosed to any patient until the study had concluded, and all study participants were discouraged from discussing with one another their involvement in the trial. Finally, the clinical researchers strictly abided by the study’s random design and interacted with the patients in each group with as few differences as possible. Blinding was maintained at the level of outcome assessment. The individuals performing laboratory blood analyses, data management, and statistical analysis were independent of the clinical component of the study, and were not provided with any information that may reveal treatment allocation details.

### Statistical analysis

Statistical analyses were performed with SAS9.3 software (SPSS, Inc., Chicago, IL, USA). Categorical data were reported as counts with the percentage of the total and continuous data as a mean with standard deviation, or as a median with 95% confidence interval (CI) or interquartile range (IQR). The χ^2^ test or Fisher’s exact test was used for comparisons of categorical data. Because serum levels of hs-CRP, IL-6, TNF𝛼, VCAM-1, sCD40L, MCP-1, and MMP-9 were measurement data, an independent Student’s *t*-test was used for the analysis of intergroup differences when the date meet the normal distribution; Wilcoxon rank-sum test was used when the distribution of levels of the data was non-normality. In addition, comparison of inflammatory markers levels within the group be conducted by paired t-test or paired Wilcoxon rank-sum test. Wilcoxon rank-sum test was used to the analysis of intergroup differences of total angina scores because the distribution of levels of the data was non-normality. All tests were two tailed and a statistical probability of <0.05 was considered significant.

## Electronic supplementary material

Supplementary information
