# Supplementary material for: Sodium tanshinone IIA sulfate adjunct therapy reduces high-sensitivity C-reactive protein level in coronary artery disease patients: a randomized controlled trial
Source: Sci Rep. 2017 Dec 12;7:17451. doi: 10.1038/s41598-017-16980-4 (PMC5727111; doi:10.1038/s41598-017-16980-4)
Supplement: Supplementary file 1 — Supplementary information [file 41598_2017_16980_MOESM1_ESM.pdf]

Sodium tanshinone IIA sulfate adjunct therapy reduces high-sensitivity C-reactive protein level in coronary artery disease patients: a randomized controlled trial

Siming Li[1,+], Yang Jiao[1,2,+], Hanjay Wang[3], Qinghua Shang[1], Fang Lu[4], Li Huang[5], Jiangang Liu[1], Hao Xu[1]*, Keji Chen[1]

[1] Cardiovascular Diseases Center, Xiyuan Hospital, China Academy of Chinese Medical Sciences, Beijing 100091, China.
[2] Graduate School, Beijing University of Chinese Medicine, Beijing 100029, China.
[3] Department of Cardiothoracic Surgery, Stanford University, Palo Alto, CA 94304, USA.
[4] Institute of Clinical Pharmacology, Xiyuan Hospital, China Academy of Chinese Medical Sciences, Beijing 100091, China.
[5] Integrative Cardiology Department, China-Japan Friendship Hospital, Beijing 100029, China.
*Correspondence should be addressed to Hao Xu, xuhaotcm@hotmail.com.
[+] These authors contributed equally to this work.

Supplement Table 1. Calculation of angina risk score

Angina Frequency	
<1 episode per week	0 points
1-6 episodes per week	2 points
1-3 episodes per day	4 points
>4 episodes per day	6 points
Angina Duration, per episode	
No angina	0 points
≤5 minutes	2 points
Between 5-10 minutes	4 points
≥10 minutes	6 points
Angina Intensity	
No angina	0 points
Relieved with rest. Does not affect daily life.	2 points
Relieved with medication. Can endure moderate exercise.	4 points
Angina frequently induced with activities of daily life (e.g. dressing, eating, walking)	6 points
Nitroglycerin usage	
<1 pill per week	0 points
1-4 pills per week	2 points
5-9 pills per week	4 points
≥10 pills per week	6 points

Total angina score equals the sum of the component scores. Minimum score = 0 points. Maximum score = 24 points.
